# Optimized Isolation of White Tea Infusion Micro-Nanoparticles and Stability Mechanism: A Composition–Structure–Stability Perspective

**DOI:** 10.3390/foods15081408

**Published:** 2026-04-17

**Authors:** Yuan Sun, Chuyu Ye, Fei Xu, Cheng Peng, Ying Xu, Dongfeng Wang

**Affiliations:** College of Food Science and Engineering, Ocean University of China, Qingdao 266404, China; sunyuan@stu.ouc.edu.cn (Y.S.); 1515882386@139.com (C.Y.); xuf_1029@stu.ouc.edu.cn (F.X.); pengcheng_nong@163.com (C.P.); wangdf@ouc.edu.cn (D.W.)

**Keywords:** white tea micro-nanoparticles, TOPSIS, stability, multi-spectroscopic analysis, molecular dynamics

## Abstract

White tea infusion micro-nanoparticles (WTMPs) are important for colloidal stability, but the optimal strategy for their isolation and the mechanisms underlying their stability remain unclear. Here, a multi-indicator TOPSIS strategy was used to optimize ultrafiltration–centrifugation, and the best condition was identified as a 100 kDa membrane, 3000× *g*, and 20 min. The isolated WTMPs were not merely a concentrated form of white tea colloids (WTCs), but a selectively enriched fraction with remodeled composition, more regular morphology, more ordered intermolecular organization, and improved environmental stability. In particular, gallic acid (GA) was enriched, whereas caffeine (CAF) decreased markedly after isolation. Spectroscopy and molecular dynamics simulations further suggested that GA and CAF played different roles in the protein–polysaccharide network: GA was more favorable for cooperative interfacial stabilization, whereas CAF behaved more like a locally associated ligand. Overall, these results support a composition–structure–stability relationship for WTMPs and provide mechanistic insight into the selective enrichment of GA and the enhanced stability of the isolated micro-nanoparticle fraction.

## 1. Introduction

Tea is one of the most widely consumed natural beverages worldwide. Among the six major tea categories in China, white tea has attracted increasing attention because of its minimal processing, mild sensory profile, and relatively high retention of bioactive constituents [1]. In addition to its chemical composition, a white tea infusion should also be viewed as a complex colloidal system rather than a simple aqueous extract. It contains suspended particles, colloidal components, and truly dissolved substances in dynamic equilibrium. In this system, proteins, polysaccharides, polyphenols, caffeine, and mineral elements can interact through hydrogen bonding, hydrophobic interactions, van der Waals forces, and electrostatic effects [2]. These interactions promote the formation of micro-nanoscale composite particles. Such particles are closely related to infusion stability, sensory quality, and the retention of bioactive compounds [3].

The stability of the tea infusion is important for both quality control and product development. When the colloidal balance is disturbed, the infusion may become turbid, separate into phases, lose color quality, and show reduced retention of functional components. Previous studies on green tea and black tea have shown that natural tea nanoparticles are closely related to haze formation, flavor expression, and biological activity. Their size distribution, morphology, and composition are also linked to infusion quality [4,5,6]. These findings suggest that understanding the formation and stability of tea-derived nanoparticles is important for explaining the behavior of tea infusions.

However, research on nanoparticles in white tea infusion remains limited [7]. First, there is still no widely accepted strategy for the separation and purification of white tea infusion nanoparticles, especially one that is oriented toward stability. Objective multi-criteria methods for process optimization are also rarely used. Second, most existing studies only focused on broad compositional descriptions and did not systematically connect the separation results with the physicochemical properties, molecular organization, and environmental stability. As a result, it is still unclear whether the isolated nanoparticles are simply concentrated colloidal fractions or a selectively enriched population with distinct structural and compositional features. Third, the molecular basis of particle formation, selective component retention, and enhanced stability remains poorly understood.

Recent studies on protein–polysaccharide–polyphenol ternary systems provide a useful framework for understanding the stabilization of multicomponent colloidal particles. In these systems, polyphenols can participate in cooperative assembly through hydrogen bonding, hydrophobic interactions, and van der Waals forces. At the same time, electrostatic interactions can further regulate colloidal organization and stability under different environmental conditions [8,9]. This perspective suggests that the stability of tea-derived nanoparticles should be interpreted from a composition–structure–stability relationship rather than from isolated binding events alone.

Therefore, this study proposed that an optimized ultrafiltration–centrifugation process would not simply concentrate pre-existing colloids in white tea infusion. Instead, it would selectively enrich a micro-nanoparticle fraction with remodeled composition, stronger intermolecular organization, and better environmental stability. Based on this idea, the objectives of this study were to: (i) establish a multi-indicator and stability-oriented strategy for optimizing the isolation of white tea infusion micro-nanoparticles (WTMPs) from white tea colloid (WTC); (ii) compare the composition, morphology, structural features, and environmental stability of the isolated nanoparticle fraction with those of the parent tea infusion colloidal system; and (iii) combine spectroscopy and molecular dynamics simulation to clarify the molecular basis of micro-nanoparticle stability. This study aims to establish a composition–structure–stability framework for white tea infusion micro-nanoparticles and provide a theoretical basis for improving white tea quality and developing white tea-based functional products.

## 2. Materials and Methods

### 2.1. Materials and Reagents

White tea was sourced from Yunnan Tongrui Tea Co., Ltd. (Lvchun, China). Folin–Ciocalteu reagent and bovine serum albumin (BSA) were purchased from Solarbio (Beijing, China). Anthrone, sulfuric acid, hydrochloric acid, sodium hydroxide, potassium chloride, sodium bicarbonate, ammonium carbonate, anhydrous D-glucose, gallic acid and anhydrous sodium carbonate (all analytical grade) were purchased from Sinopharm Chemical Reagent Co., Ltd. (Shanghai, China). Gallic acid and caffeine (HPLC grade) were purchased from Shanghai Yuanye Bio-Technology Co., Ltd. (Shanghai, China). Potassium bromide (spectroscopic grade) was purchased from Aladdin Biochemical Technology Co., Ltd. (Shanghai, China). Pectin (analytical grade) was purchased from Shanghai Macklin Biochemical Technology Co., Ltd. (Shanghai, China). Ultrafiltration membranes (100 kDa and 30 kDa MWCO, polyethersulfone) were purchased from Merck Millipore (Billerica, MA, USA). Unless otherwise noted, all reagents were of analytical grade and ultrapure water was used throughout.

### 2.2. Preparation of White Tea Infusion

White tea was ground into powder and passed through a 60-mesh sieve. The tea infusion was prepared at a tea-to-water ratio of 1:40 (*w*/*v*) by heating in a water bath at 90 °C for 30 min. The mixture was then filtered through a sieve to remove large solid particles, cooled to 40 °C, and passed through a 0.45 μm membrane filter to obtain WTCs. The resulting WTCs were then subjected to ultrafiltration–centrifugation for WTMP isolation. The detailed optimization procedure is described in Section 2.4.

### 2.3. Determination of the Colloidal Properties and Conductivity

WTMPs were characterized by dynamic light scattering following the previous method [10] with minor modifications. Measurements of Z-Ave, count rate, polydispersity index (PDI), and zeta potential were performed on a Zetasizer Nano ZS90 (Malvern Instruments, Malvern, UK). Dispersions were equilibrated at 25 °C for 120 s prior to analysis, and the viscosity and refractive index in the software were set to 0.8872 cP and 0.135, respectively. Conductivity of WTMP dispersions was determined at 25 °C [11] using a benchtop meter (DDSJ-308F, Shanghai INESA Scientific Instrument Co., Ltd., Shanghai, China).

### 2.4. Optimization of the Extraction Method for Micro-Nanoparticles

The separation procedure for isolating micro-nanoparticles from green tea [5] was adopted with modifications. Briefly, 15 mL of microfiltered white tea infusion was subjected to ultrafiltration using membranes with molecular weight cut-offs (MWCO) of 30 and 100 kDa, followed by centrifugation at 2000×, 3000×, 4000×, 5000×, or 6000× *g* for 10, 20, or 30 min. Ultrafiltration membranes with MWCOs of 30 and 100 kDa were selected as two representative separation conditions to compare relatively tighter and looser retention ranges within a practically feasible experimental window. Since the target WTMPs had hydrodynamic sizes on the order of hundreds of nanometers, both membranes were expected to effectively retain the particle fraction while allowing for a comparison of differences in purification behavior and colloidal properties. In total, 30 separation conditions were tested. The retentates containing WTMPs obtained under each condition were diluted with ultrapure water to 10 mL for subsequent analysis.

Optimization of the separation conditions was conducted using the TOPSIS method [12]. The parameters of hydrodynamic diameter, scattering intensity, PDI, zeta potential, and conductivity were selected as decision criteria. These five criteria were selected because they jointly reflect the key quality dimensions of the isolated WTMP fraction, namely particle retention, dispersion uniformity, colloidal stability, and purification efficiency. Specifically, Z-Ave was used to characterize the overall particle size level, since excessive size growth usually indicates aggregation. Count rate was included to reflect the abundance of dispersed particles in the retentate. PDI was used to evaluate distribution uniformity, with lower values indicating a more homogeneous colloidal system. The absolute value of zeta potential was used as an indicator of electrostatic stabilization because larger absolute values are generally associated with stronger interparticle repulsion. Conductivity was included to reflect the removal of low-molecular-weight charged solutes and thus the purification degree of the isolated fraction. The TOPSIS procedure involved six computational steps.

Preparation stage: *A* decision matrix (*A_ij_*) was constructed for ranking.

(1) $$A_{ij}=\left[\begin{array}{cccc} x_{11} & x_{12} & \cdots & x_{1n}\\ x_{21} & x_{22} & \cdots & x_{2n}\\ \vdots & \vdots & \ddots & \vdots \\ x_{m1} & x_{m2} & \cdots & x_{mn} \end{array}\right]$$

*m* represents the number of samples, and *n* represents the number of decision criteria.

Step 1. Normalize the decision matrix using Equation (2).

(2) $$Z_{ij}=\frac{X_{ij}}{\sqrt{\sum_{i=1}^{m}{\left(X_{ij}\right)}^{2}}}$$

Here, *Z_ij_* represents the normalized value, while *X_ij_* is the actual measured value.

Step 2. Calculate the weighted normalized decision matrix (U) using Equation (3). The weights assigned to the decision criteria of hydrodynamic diameter, scattering intensity, PDI, absolute value of zeta potential, and conductivity were 0.15, 0.20, 0.25, 0.15, and 0.25, respectively. These weights were determined through expert consultation based on the practical objective of obtaining a stable and well-defined nanoparticle fraction, with relatively higher weights assigned to PDI and conductivity because dispersion uniformity and purification efficiency were considered especially important.

(3) $$U_{ij}=Z_{ij}\times w_{ij}$$

Here, *U_ij_* represents the weighted normalized value, *Z_ij_* is the normalized value, and *w_ij_* is the weight assigned to each criterion.

Step 3. Determine the positive ideal solution (*A*^+^) and the negative ideal solution (*A*^−^) using the following equations.

(4) $$A^{+}=\left\{U_{1}^{+},U_{2}^{+},U_{3}^{+},\ldots ,\;U_{n}^{+}\right\}\;\left(maximum\;values\right)$$

(5) $$A^{-}=\left\{U_{1}^{-},U_{2}^{-},U_{3}^{-},\ldots ,U_{n}^{-}\right\}\;\left(minimum\;values\right)$$

Step 4. Calculate the distance (*d*) of each alternative from the positive and negative ideal solutions using Equations (6) and (7).

(6) $$d_{i}^{+}=\sqrt{\sum_{i=1}^{n}{\left(U_{ij}-U_{j}^{+}\right)}^{2}}$$

(7) $$d_{i}^{-}=\sqrt{\sum_{i=1}^{n}{\left(U_{ij}-U_{j}^{-}\right)}^{2}}$$

Step 5. Calculate the closeness coefficient (*C*) of each alternative using Equation (8).

(8) $$C=\frac{d_{i}^{-}}{d_{i}^{+}+d_{i}^{-}}$$

Step 6. Select the alternative with the highest *C* value as the optimal option.

### 2.5. Analysis of the Chemical Composition of WTCs and WTMPs

#### 2.5.1. Pretreatment of WTCs and WTMPs

After pre-freezing the WTCs and WTMPs at −80 °C, the samples were subjected to vacuum drying for 48 h to obtain powdered WTCs and WTMPs. Prior to measurement, the powders were dissolved in ultrapure water to a concentration of 1 mg/mL for subsequent use.

#### 2.5.2. Determination of Total Polysaccharide Content

Total polysaccharides were quantified by the anthrone–sulfuric acid assay [13] with minor modifications. Briefly, 1.0 mL of sample was combined with 4.0 mL anthrone–sulfuric acid reagent (2 mg/mL), heated at 100 °C in a boiling water bath for 20 min, and cooled to ambient temperature. Absorbance was measured at 620 nm on a UV–Vis spectrophotometer (UV-2550, Shimadzu, Kyoto, Japan). Quantification was based on a glucose calibration curve.

#### 2.5.3. Determination of Protein Content

The protein content was determined using the method [14] with slight modifications. Briefly, 1 mL of sample solution was mixed with 4 mL of Bradford reagent (prepared by dissolving 50 mg of Coomassie Brilliant Blue G-250 in 25 mL of 95% ethanol and 50 mL of 85% phosphoric acid, followed by dilution with ultrapure water to a final volume of 500 mL) and incubated at 25 °C for 5 min. The absorbance was then measured at 595 nm using a UV–visible spectrophotometer. A calibration curve was constructed with bovine serum albumin (BSA) as the standard.

#### 2.5.4. Determination of Tea Polyphenol Content

The tea polyphenol content was determined using the Folin–Ciocalteu method [15]. Briefly, 1 mL of the sample was mixed with 5 mL of 10% (*v*/*v*) Folin–Ciocalteu reagent and left at 25°C for 5 min. Then, 4 mL of 7.5% (*w*/*v*) sodium carbonate solution was added, and the reaction was allowed to proceed at 25 °C for 60 min. Absorbance was measured at 765 nm using a UV–visible spectrophotometer. Total phenolics were quantified using a gallic acid standard curve.

#### 2.5.5. Determination of Gallic Acid and Caffeine Content

The contents of gallic acid and caffeine were determined using high-performance liquid chromatography (HPLC) with a C_18_ column (Agilent 1260 Infinity, Agilent Technologies, Santa Clara, CA, USA) [16]. Calibration curves were constructed by plotting the concentration of each individual standard (*x*-axis) against its peak area (*y*-axis) at the same retention time. Standard solutions were prepared at 50–1000 μg/mL for GA and 50–1000 μg/mL for CAF. HPLC conditions were as follows: detection wavelength of 278 nm, flow rate of 1 mL/min, column temperature of 30 °C, and total run time of 30 min.

### 2.6. Morphological Observation of WTCs and WTMPs

For transmission electron microscope (TEM) analysis, a drop of sample suspension was placed onto a 300-mesh copper grid and negatively stained with 5% uranyl acetate. Excess liquid was removed with filter paper, and the grid was dried under an infrared lamp. The specimens were then examined with a transmission electron microscope (JEOL JEM-1400Plus, Tokyo, Japan) operated at 120 kV, and micrographs were recorded to assess the morphology of WTMPs.

For scanning electron microscope (SEM) observation, 5 μL of WTC or WTMP suspension was deposited onto a clean copper stub and air-dried at room temperature. The dried samples were sputter-coated with a thin layer of gold and subsequently examined using a scanning electron microscope (EVO10, ZEISS, Baden-Württemberg, Germany) to visualize the surface morphology and particle size distribution [17]. Representative images were acquired from selected regions of interest.

### 2.7. FTIR Analysis of WTCs and WTMPs

Samples were thoroughly mixed with potassium bromide (KBr) at a ratio of 1:100 (*w*/*w*), finely ground, and pressed into transparent pellets in a Fourier transform infrared spectrometer (Nicolet iS10, Thermo Scientific, Waltham, MA, USA). Spectra were recorded in the range of 4000–400 cm^−1^ with a resolution of 4 cm^−1^, and 32 scans were accumulated for each sample [18].

### 2.8. Stability Evaluation of WTCs and WTMPs

#### 2.8.1. Effect of Temperature

The effect of temperature on WTCs and WTMPs was evaluated using a Malvern Nano ZS90 particle size analyzer. Samples were subjected to heating and cooling cycles within the range of 10–70 °C, and Z-Ave was measured at 10 °C intervals during both the heating and cooling processes.

#### 2.8.2. Effect of pH

The pH of the WTC and WTMP suspensions was adjusted stepwise to the desired values (pH 3–12) by dropwise addition of 0.1 M HCl or 0.1 M NaOH under gentle stirring. After equilibration at 25 °C for 10 min, the final pH values were verified using a calibrated pH meter. The colloidal properties were then determined at the measured pH values. All measurements were performed in triplicate.

#### 2.8.3. Effect of Cold Storage and Freeze–Thaw Cycles

Cold storage: Aliquots (20 mL) of WTCs and WTMPs were stored at 4 °C. Samples were collected every other day over a 7-day period, and Z-Ave was determined as described above.

Freeze–thaw cycles: Aliquots (20 mL) of WTCs and WTMPs were stored at −20 °C and subjected to repeated freeze–thaw cycles. Samples were thawed every other day over a 7-day period, and the Z-Ave values were measured as described above.

### 2.9. UV–Visible Absorption Spectroscopy

The UV–visible absorption spectra of BSA in the absence and presence of PEC/GA/CAF were recorded using a UV–Vis spectrophotometer (UV-2550, Shimadzu, Tokyo, Japan) over the wavelength range of 230–360 nm at 25 °C. This spectral window was selected because it covers the main absorption region relevant to the aromatic residues of BSA and the ligand-induced spectral changes discussed in the present study. The BSA concentration was fixed at 1.0 × 10^−4^ mol/L. PEC concentrations ranged from 2 to 8 × 10^−6^ mol/L, and GA or CAF concentrations ranged from 1 to 9 × 10^−7^ mol/L.

### 2.10. Fluorescence Spectroscopy

The fluorescence spectra were measured according to a method [19] with minor modification. In brief, BP was used as the reference system (with BSA at 1.0 × 10^−4^ mol/L and PEC at 4.0 × 10^−6^ mol/L) to which GA/CAF solutions at concentrations of 0.1–10 × 10^−7^ mol/L were added. Fluorescence spectra of the mixtures were recorded using a fluorescence spectrophotometer (F-4600, Hitachi, Tokyo, Japan) at 25 °C with an excitation wavelength of 280 nm. Emission spectra were collected over 300–450 nm. The bimolecular quenching rate constant (Ksv) and the dynamic quenching constant (Ka) were determined from the fluorescence intensity at the maximum emission wavelength.

### 2.11. Circular Dichroism Spectroscopy

The circular dichroism spectra were measured according to a method [12] with minor modification. Briefly, BP complex solutions were prepared as described above (BSA, 1.0 × 10^−4^ mol/L; PEC, 4.0 × 10^−6^ mol/L). The concentrations of the GA and CAF stock solutions were each set to 1.0 × 10^−6^ mol/L. BP was then mixed with either the GA or CAF solutions at volume ratios of 1:1, 1:2, 1:5, 1:10, 1:20, and 1:50. These freshly prepared aqueous samples were analyzed using a circular dichroism spectropolarimeter (J-1500, JASCO, Tokyo, Japan) to assess changes in protein conformation. The sample chamber was continuously purged with argon. Spectra were recorded over 190–260 nm with a scan step of 0.5 nm.

### 2.12. Molecular Docking and MD Simulations

#### 2.12.1. MD Simulations

The amino acid sequence of BSA was obtained from the UniProt database (https://www.uniprot.org/uniprotkb/O15399/entry (accessed on 15 September 2025)), and its 3D structure was retrieved from the RCSB Protein Data Bank (PDB ID: 8WDD). The protein structure was preprocessed using PyMOL (version 2.5.4), including the removal of water molecules and hydrogen addition. Ligand structures were obtained from the PubChem database (https://pubchem.ncbi.nlm.nih.gov/ (accessed on 20 September 2025)). Molecular docking was performed using the CB-DOCK2 server (https://cadd.labshare.cn/cb-dock2/php/blinddock.php (accessed on 27 September 2025)), which employs artificial neural network-based cavity detection and uses AutoDock Vina (version 1.5.7) for docking [20]. Docked complexes were ranked based on binding energy, and interaction forces and binding sites were further analyzed to select structures for subsequent MD simulations. Protein–ligand interactions were analyzed using the PLIP web server (https://plip-tool.biotec.tu-dresden.de/plip-web (accessed on 28 September 2025)), and 3D conformations of complexes were visualized with PyMOL.

All-atom MD simulations of the selected protein–ligand complexes were performed using GROMACS 2023.3 with the AMBER force field [21]. Hydrogen atoms were added with the pdb2gmx module, and the systems were solvated in a truncated cubic TIP3P water box with a 10 Å margin [19]. Na^+^ and Cl^−^ counter-ions were introduced to neutralize the system charges. Topology and parameter files were generated for subsequent simulations. Energy minimization was first conducted using the steepest descent algorithm with an initial step size of 0.01 nm and a force tolerance of 1000 kJ/(mol·nm). Equilibration was performed in two stages: (i) a 100 ps NVT simulation, during which the temperature was gradually increased from 0 K to 310.15 K, allowing the solvent molecules to distribute uniformly, and (ii) a 100 ps NPT simulation with the Berendsen barostat to equilibrate pressure to 1 bar [22].

Production MD simulations were run for 100 ns [23]. All bonds involving hydrogen atoms were constrained using the LINCS algorithm, with an integration time step of 2 fs. Electrostatic interactions were calculated with the particle mesh Ewald (PME) method using a cutoff of 1.2 nm. The cutoff for non-bonded interactions was set to 10 Å, and neighbor lists were updated every 10 steps. The resulting trajectories were corrected for periodic boundary conditions and analyzed for root mean square deviation (RMSD), number of hydrogen bonds, solvent-accessible surface area (SASA), and secondary structure evolution.

#### 2.12.2. MM/GBSA Binding Free Energy Calculations

The binding free energy between protein and ligands was calculated using the molecular mechanics/generalized Born surface area (MM/GBSA) approach [24,25,26]. The calculation was based on the following formula:

(9) $${\Delta G}_{bind}={\Delta G}_{complex}-\left({\Delta G}_{receptor}+{\Delta G}_{ligand}\right)$$

(10) $${\Delta G}_{bind}={\Delta E}_{internal}+{\Delta E}_{VDW}+{\Delta E}_{elec}{+\Delta G}_{GB}+{\Delta G}_{SA}$$

In Equations (9) and (10), Δ*E*_internal_ represents the internal energy, Δ*E_VDW_* denotes van der Waals interactions, and Δ*E*_elec_ corresponds to electrostatic interactions. The internal energy consists of bond energy (*E*_bond_), angle energy (*E*_angle_), and torsional energy (*E*_torsion_). Δ*G*_GB_ and Δ*G*_SA_ collectively represent the solvation free energy, where Δ*G*_GB_ is the polar solvation free energy and Δ*G*_SA_ is the nonpolar solvation free energy. For Δ*G*_GB_, the generalized born (GB) model was applied (igb = 2) [27]. The nonpolar solvation free energy (Δ*G*_SA_) was calculated as the product of surface tension (γ) and SASA, using the relation Δ*G*_SA_ = 0.0072 × ΔSASA. The entropic contribution (−TΔS) was neglected in this study due to its high computational cost and limited accuracy [25].

### 2.13. Data Processing

All experiments were performed in triplicate, and three sets of parallel data were obtained. Data processing and plotting were carried out using Excel 2021 and OriginPro 2022. Statistical significance was analyzed with IBM SPSS Statistics 26. Different letters were used to indicate significant differences between groups (*p* < 0.05, unless otherwise stated). Differences between WTCs and WTMPs were evaluated using an independent samples *t*-test. Statistical significance between WTCs and WTMPs was further supported by reporting the corresponding *p* values for the main comparisons.

## 3. Results and Discussion

### 3.1. Effect of MWCO on the Properties of Micro-Nanoparticles

The ultrafiltration–centrifugation method uses centrifugal force to create transmembrane pressure. This pressure pushes solvent and small solutes through a semipermeable membrane with a defined MWCO. Components larger than the MWCO are retained. They become concentrated in the retentate. Smaller molecules pass through and end up in the permeate. In white tea infusion colloids (WTCs), the micro-nanoparticles are mainly protein–polyphenol complexes. Their hydrodynamic size is much larger than the MWCO scale. As a result, they are efficiently retained and enriched. Free small molecules are removed at the same time, which include catechin monomers, alkaloids, free amino acids, organic acids, and inorganic ions. To evaluate the resulting WTMPs, we used several common colloidal indicators. These included Z-Ave, count rate, PDI, conductivity, and zeta potential. Z-Ave reflects the particle size, the count rate indicates particle abundance, PDI describes how broad the size distribution is, and conductivity gives a quick read on ion removal. Lower values suggest better clearance. Zeta potential is another stability marker. A larger absolute value usually means less aggregation and better stability.

#### 3.1.1. Effect of Centrifugal Force and Centrifugation Time on the Characteristics of Micro-Nanoparticles Using a 30 kDa Membrane

**Figure 1:**
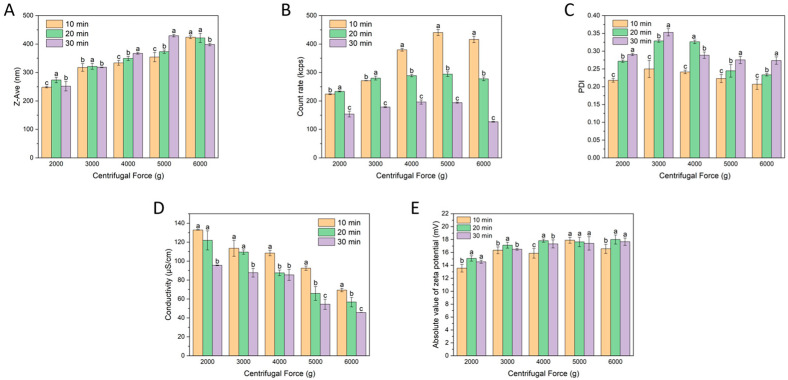
Variations in colloidal properties of WTMPs using a 30 kDa ultrafiltration membrane: (**A**) Z-Ave, (**B**) count rate, (**C**) PDI, (**D**) conductivity, and (**E**) zeta potential. Different lowercase letters represent a significant difference at *p* < 0.05.

Under the 30 kDa membrane condition, the colloidal properties of the isolated particles changed markedly with centrifugal force and centrifugation time. This finding indicates that micro-nanoparticle isolation is highly sensitive to the operating conditions. As shown in Figure 1A, Z-Ave generally increased with increasing centrifugal force. This trend should be interpreted in the context of ultrafiltration–centrifugation rather than simple sedimentation alone. Under these conditions, increasing centrifugal force may promote the preferential loss of smaller or more weakly associated particles through the membrane, thereby enriching the retained fraction in relatively larger particle populations [28]. In contrast, the effect of centrifugation time was not consistent across force levels. At 2000 g and 6000 g, the Z-Ave after 30 min was lower than that after 10 min, whereas the opposite trend was observed at 4000 g and 5000 g. This non-uniform response suggests that particle migration and retention were influenced not only by membrane exclusion, but also by differences in particle size, density, and structural stability during centrifugation.

The count rate showed a similar condition-dependent pattern (Figure 1B). At 5000 g for 10 min, the count rate exceeded 400 kcps, indicating effective enrichment of dispersed particles. However, further increases in force or duration did not improve the signal and instead caused a decline. This result suggests that under higher centrifugal force, some smaller or more weakly associated colloidal particles may dissociate or be more easily removed, while part of the dissociated components may further reorganize or reassociate with larger retained particles, leading to relatively larger colloidal assemblies. This interpretation is consistent with previous reports on tea colloids [4]. In comparison, the PDI values remained within 0.22–0.35 (Figure 1C), indicating that the isolated particles still showed a relatively narrow size distribution under all tested conditions.

Conductivity and zeta potential further reflected the separation effect of the 30 kDa membrane (Figure 1D,E). With increasing centrifugal force, conductivity decreased markedly, while the absolute value of zeta potential increased. These results indicate that ultrafiltration–centrifugation effectively removed small charged solutes from WTCs and enriched a colloidal fraction with improved electrostatic stability [29]. Nevertheless, the overall results also showed that stronger centrifugation did not always lead to better isolation quality. Instead, the 30 kDa system displayed a trade-off between particle retention, particle abundance, and colloidal stability. Therefore, the quality of WTMP isolation could not be judged by any single parameter alone. In addition, these findings made it necessary to further compare membrane cut-off conditions.

#### 3.1.2. Effect of Centrifugal Force and Centrifugation Time on the Characteristics of Micro-Nanoparticles Using a 100 kDa Membrane

**Figure 2:**
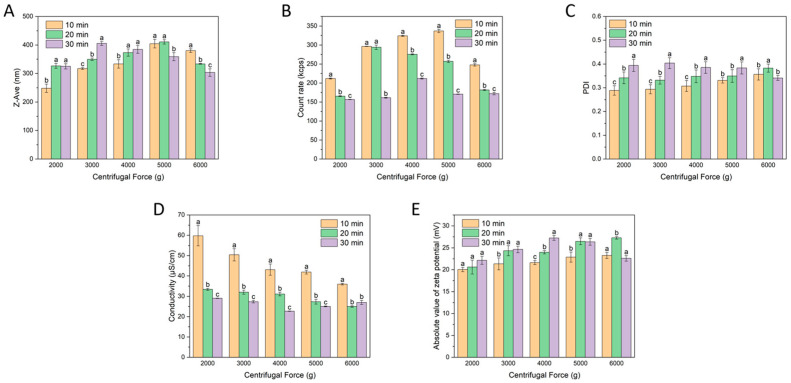
Variations in colloidal properties of WTMPs using a 100 kDa ultrafiltration membrane: (**A**) Z-Ave, (**B**) count rate, (**C**) PDI, (**D**) conductivity, and (**E**) zeta potential. Different lowercase letters represent a significant difference at *p* < 0.05.

The principle of separating micro-nanoparticles using a 100 kDa membrane is the same as that of the 30 kDa membrane, with the difference lying in the MWCO. As shown in Figure 2A, the overall trend was consistent with the results obtained using the 30 kDa membrane, but the particle size of the final retentate was slightly larger. Although the 30 and 100 kDa membranes differed in MWCO, the size differences of the retained particle fractions were not very large. This is likely because the target WTMPs had hydrodynamic sizes on the order of hundreds of nanometers, which are already much larger than the nominal separation scale of both membranes. Therefore, both membranes effectively retained the main particle fraction, whereas the major difference between them was reflected more in purification behavior than in a large shift in particle size. The variations in scattering intensity shown in Figure 2B–E were also generally consistent with the corresponding results in Figure 1. In addition, the conductivity further decreased compared with the 30 kDa condition, suggesting that the larger pore size enabled more efficient removal of charged small molecules, thereby improving the purification of WTMPs.

Taken together, these results indicate that the 100 kDa membrane maintained effective retention of WTMPs while allowing for more efficient removal of low-molecular-weight components. Compared with the 30 kDa membrane, the 100 kDa system appeared more favorable for obtaining a purified nanoparticle fraction with reduced interference from free small solutes. However, the effects of centrifugal force and time were still not completely linear, and the optimal isolation condition could not be determined from a single indicator alone.

#### 3.1.3. Integrated Evaluation Identifies the Optimal Isolation Condition

The results under the two membrane conditions showed that WTMP isolation involved several trade-offs. Lower conductivity indicated more efficient removal of free charged solutes. A larger absolute value of zeta potential suggested better electrostatic stability. At the same time, particle size, particle abundance, and size distribution also needed to be considered. Therefore, a comprehensive evaluation was required to identify the best isolation condition.

To address this issue, the TOPSIS method was used for multi-criteria evaluation [30], Z-Ave, count rate, PDI, conductivity, and zeta potential were integrated into one evaluation framework (Table 1). In this analysis, lower Z-Ave, PDI, and conductivity values were considered favorable, whereas higher count rate and a larger absolute value of zeta potential were considered favorable. This approach allowed for all tested conditions to be compared at the same level and provided a more balanced evaluation of isolation quality.

As shown in Table 1, the combination of a 100 kDa membrane, 3000× *g*, and 20 min was identified as the optimal condition to isolate the WTMPs. Notably, under this setting, Z-Ave was 349 nm and PDI was 0.34—neither the best single-parameter values—but the zeta potential was higher in magnitude and the conductivity was lower, indicating more effective removal of charged small molecules. These features collectively justified this condition as the best overall separation scheme.

This result is important for two reasons. First, it confirms that stronger processing does not necessarily produce a better WTMP fraction. The optimal condition was defined by overall balance, rather than by the extreme value of one parameter. Second, it shows that the 100 kDa membrane was more suitable than the 30 kDa membrane for isolating a purified and stable nanoparticle fraction from WTCs. Based on this result, the WTMPs obtained under the 100 kDa-3000× *g*-20 min condition were used for all subsequent analyses. More importantly, the optimized process did not simply concentrate the original tea colloids. Instead, it generated a more stable and better-defined WTMP fraction, which provided the basis for the following analyses of composition, structure, and environmental stability. Nevertheless, the present optimization was conducted at laboratory scale, and further validation will be needed to assess the reproducibility and scalability of this ultrafiltration–centrifugation procedure in practical applications.

### 3.2. Optimized Isolation Selectively Remodels the Composition of WTMPs

After the optimized separation procedure, the major categories of components in the WTMPs remained broadly similar to those in the parent WTCs. Proteins, polysaccharides, polyphenols, and mineral elements were still the main constituents. This result indicates that the isolated WTMPs were not an artificial fraction generated during processing, but a representative micro-nanoparticle fraction derived from the original tea colloidal system. However, although the major component classes were retained, their relative contents changed noticeably after isolation. These compositional changes are important because they help explain how the optimized process reshaped the nanoparticle fraction and why the resulting WTMPs showed improved stability.

As shown in Table 2, proteins remained the dominant macromolecular component in WTMPs and accounted for about 41% of the total composition, slightly higher than the 38% observed in WTCs. This result suggests that proteins were preferentially retained during ultrafiltration–centrifugation and likely formed an important structural basis of the nanoparticle fraction. In contrast, total polysaccharides showed only a slight decrease, and the total tea polyphenols showed a moderate decrease. Even so, proteins, polysaccharides, and tea polyphenols still accounted for more than 90% of the total composition, which is consistent with the major compositional features of tea infusion colloids. Therefore, the optimized isolation did not fundamentally change the nature of the system, but instead enriched a compositionally related nanoparticle fraction.

More importantly, several small-molecule components showed clear selective changes. Among them, caffeine showed the most obvious decrease, with its content reduced to about two-thirds of that in the original WTCs. This finding is notable because caffeine has been reported to participate in tea precipitation through interactions with polyphenols in tea systems [31]. Its reduction may therefore weaken aggregation-promoting interactions and contribute to improved micro-nanoparticle stability. In contrast, gallic acid (GA) increased markedly after isolation. This result suggests that GA was not removed simply as a free small molecule, but was more likely retained through association with the nanoparticle matrix. In other words, the optimized process did not just remove low molecular weight compounds indiscriminately. Instead, it appeared to selectively preserve small molecules that were more closely associated with the colloidal structure. Remarkably, the content of GA increased to about fourfold, which may be attributed to its small molecular weight and strong electrostatic affinity for proteins, making it less likely to be removed during ultrafiltration–centrifugation and thus exhibiting a concentration effect [32]. In fact, compositional analysis suggested that the marked increase in GA and the significant decrease in CAF were closely associated with the enhanced stability of WTMPs.

### 3.3. Optimized Isolation Produces WTMPs with a More Regular Particle Morphology

**Figure 3:**
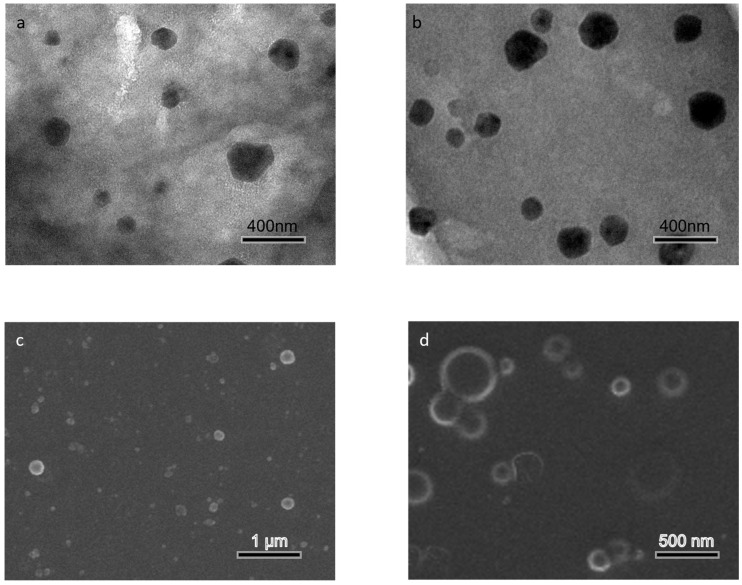
TEM and SEM images showing the representative overall morphology of WTCs and WTMPs: (**a**) TEM of WTCs, (**b**) TEM of WTMPs, (**c**) SEM of WTCs, and (**d**) SEM of WTMPs.

TEM and SEM were used to compare the overall morphology of WTCs and WTMPs. As shown in Figure 3, both samples were mainly composed of near-spherical particles, but differences were observed in particle appearance and uniformity. The particles in WTCs appeared more irregular, whereas those in the WTMPs were relatively more regular and more clearly defined. These observations, together with the DLS results, suggest that the optimized isolation enriched a particle fraction with more uniform size-related characteristics and a more regular overall morphology.

This result suggests that the optimized isolation process did not simply enrich the original colloidal particles, but also selected a nanoparticle fraction with a more ordered morphology. A more regular particle shape usually reflects a more compact and more homogeneous colloidal organization. Such a structure is generally less prone to random interparticle contact and uncontrolled aggregation. Therefore, the morphological features of WTMPs were consistent with their improved colloidal stability [33].

Together with the compositional results in Section 3.2, these observations further support the view that optimized isolation produced a selectively enriched WTMP fraction with both remodeled composition and a more organized particle structure. This also provides direct structural evidence for the enhanced stability of WTMPs compared with the parent WTC system.

### 3.4. FTIR Evidence for a More Ordered Intermolecular Organization in WTMPs

**Figure 4:**
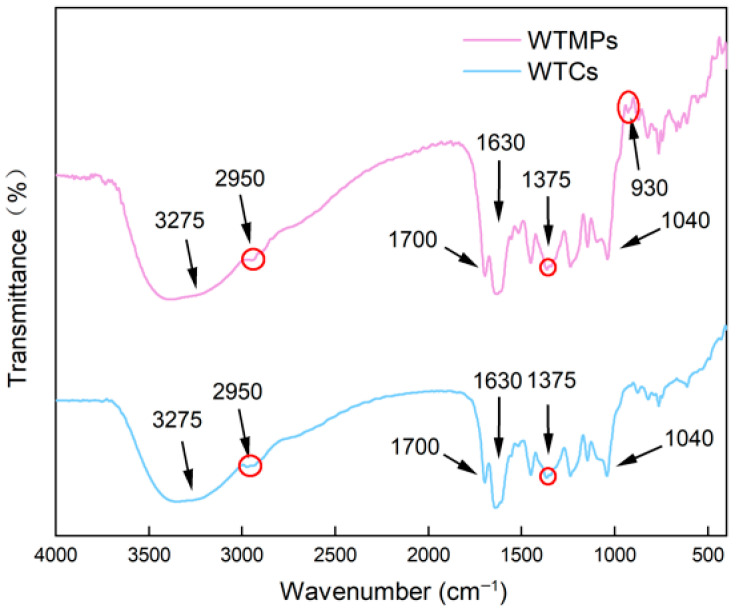
FTIR spectra of WTCs and WTMPs.

FTIR spectroscopy was used to compare the main functional-group features of WTCs and WTMPs and to evaluate possible changes in intermolecular interactions. As shown in Figure 4, both samples displayed broadly similar spectral profiles. This result indicates that the major component classes in WTMPs remained consistent with those in the parent WTC system. In other words, the optimized isolation did not create a new chemical system. Instead, it retained the main protein, polyphenol, and polysaccharide-related components of the original tea colloids [34].

In the amide region, both samples showed characteristic bands in the 1730–1625 cm^−1^ range, confirming the presence of protein related structures [35]. Compared with WTMPs, the WTC spectrum showed a subtle shoulder around 1630 cm^−1^. This feature may reflect a more complex or less uniform interaction environment in the original colloidal system. In contrast, the corresponding region in WTMPs appeared more regular, which suggests that the optimized isolation enriched a nanoparticle fraction with a more ordered protein-related organization.

Both samples also showed bands associated with polysaccharides. The signal near 1375 cm^−1^ has been linked to polysaccharide–protein assemblies, especially pectin-related interactions [36]. In addition, the 1130–990 cm^−1^ region, particularly the band around 1040 cm^−1^, confirmed the presence of glycosidic C–O–C stretching from oligo/polysaccharides [37]. A weak band near 930 cm^−1^ was also observed in WTMPs and may be related to flavonoid-associated structures.

Taken together, the FTIR results show that WTCs and WTMPs shared the same overall protein–polysaccharide–polyphenol framework. However, subtle differences in the protein-related bands suggest that the intermolecular organization in WTMPs was more concentrated and more ordered than that in WTCs. This result agrees with the compositional and morphological analyses above and further supports the view that optimized isolation selectively enriched a more structured and more stable nanoparticle fraction.

### 3.5. Optimized Isolation Improves the Environmental Stability of WTMPs

**Figure 5:**
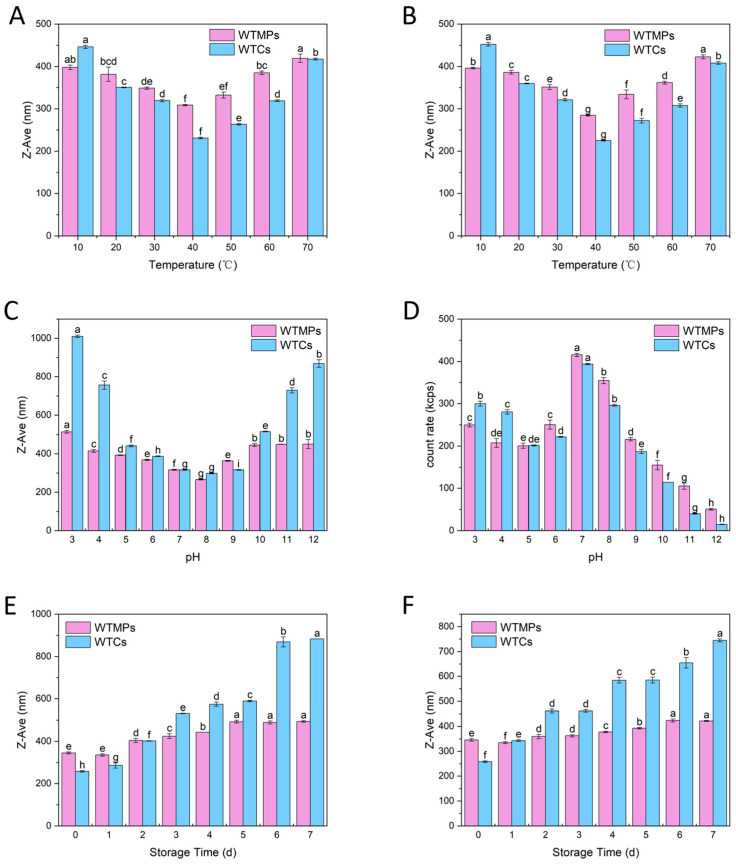
Variations in Z-Ave and count rate of WTCs and WTMPs under different conditions: (**A**) heating process, (**B**) cooling process, (**C**,**D**) measured pH values, (**E**) cold storage, and (**F**) repeated freeze–thaw cycles. Different lowercase letters represent a significant difference at *p* < 0.05.

To further evaluate whether the optimized isolation produced a more stable nanoparticle fraction, the environmental stability of WTCs and WTMPs was compared under heating, cooling, pH variation, cold storage, and freeze–thaw treatment. If the optimized process enriched a more organized and stable colloidal fraction, this advantage would be expected to appear as a smaller response to external stress. As shown in Figure 5, WTMPs consistently showed less variation than the WTCs under all tested conditions, indicating that the isolated micro-nanoparticles had better environmental stability.

#### 3.5.1. Thermal Stability Under Heating and Cooling

Tea infusions are sensitive to temperature changes, especially during cooling, which can promote haze formation and particle aggregation [38]. As shown in Figure 5A,B, the WTMPs remained much more stable than the WTCs during both heating and cooling. During heating, the Z-Ave of WTMPs changed by only 38 nm, whereas that of the WTCs changed by 227 nm. During cooling, the particle size of the WTMPs changed by 111 nm, while the WTCs showed a much larger variation of 217 nm. In both processes, the particle size of the WTCs fluctuated more strongly and increased sharply below 20 °C, whereas the WTMPs remained comparatively stable.

These results indicate that the optimized isolation produced a nanoparticle fraction with improved resistance to temperature-induced aggregation. This result is also consistent with the compositional changes described above. In particular, the decrease in caffeine and the enrichment of GA may have reduced aggregation-prone interactions and contributed to the better thermal stability of WTMPs.

#### 3.5.2. pH-Dependent Colloidal Stability

pH affects tea infusions in multiple ways—including color, taste, and chemical composition [39]. As shown in Figure 5C, the particle size of the WTCs varied from 298 to 1010 nm across pH 3–12, while the WTMPs fluctuated within a narrower range of 250–600 nm. This result indicates that WTMPs had a more stable colloidal structure over a wide pH range [40]. In both systems, particle size was smaller at pH 7–9, suggesting that this range was more favorable for colloidal stability, consistent with observations in honeysuckle tea infusions [41].

The count rate results showed a similar pattern (Figure 5D). Above pH 9, both WTCs and WTMPs showed a marked decrease in count rate, indicating that strong alkaline conditions disturbed the particle system. However, the WTMPs still remained close to 100 kcps, whereas WTCs dropped below 50 kcps. This result suggests that the particle assemblies in WTMPs were more resistant to alkaline stress, while those in the WTCs were more easily disrupted [39]. Overall, the pH results further support that the optimized isolation enriched a nanoparticle fraction with stronger intermolecular organization and better colloidal stability.

#### 3.5.3. Stability During Cold Storage and Freeze–Thaw Cycles

As shown in Figure 5E, after 7 days of cold storage, the mean particle size of the WTCs increased to above 800 nm, whereas that of the WTMPs remained around 400 nm. This result indicates that the WTMPs had much better storage stability than the parent WTC system. In contrast, the marked increase in WTC particle size suggests that prolonged refrigeration promoted further association or aggregation among colloidal components in the original tea system.

A similar advantage was observed under freeze–thaw treatment (Figure 5F). After repeated freeze–thaw cycles, the Z-Ave of WTMPs increased by less than 100 nm, indicating strong resistance to structural disruption. In both systems, the effect of freeze–thaw treatment was slightly weaker than that of continuous cold storage. However, the WTMPs still showed the smallest overall variation, confirming their superior stability under repeated physical stress.

Taken together, the stability results under temperature change, pH variation, cold storage, and freeze–thaw treatment all point to the same conclusion: the optimized isolation did not simply concentrate the original tea colloids but enriched a nanoparticle fraction with clearly improved environmental stability. This finding also agrees with the compositional, morphological, and FTIR results above, and provides functional evidence that the remodeled WTMP fraction had a more favorable basis for colloidal stability.

### 3.6. Spectroscopic Characterization of the BPG and BPC Complexes

#### 3.6.1. UV–Visible Evidence for Distinct Effects of GA and CAF on the BP Complex

**Figure 6:**
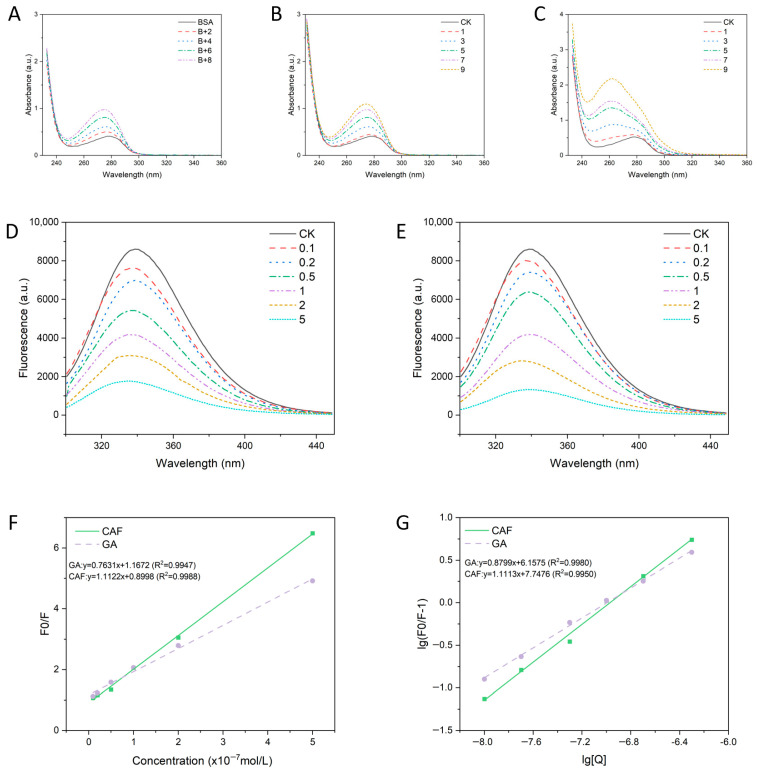
(**A**) Effects of PEC on the UV–visible spectra of BSA. (**B**) Effects of GA at different concentrations on the UV–visible spectra of the BP complex. (**C**) Effects of CAF at different concentrations on the UV–visible spectra of the BP complex. (**D**) Effects of GA at different concentrations on the fluorescence spectra of the BP complex. (**E**) Effects of CAF at different concentrations on the fluorescence spectra of the BP complex. (**F**) Stern–Volmer plots for fluorescence quenching of BP by GA and CAF. (The points in the figure represent actual measured data, and the line represents the fitted straight line. The same applies below.) (**G**) Double-logarithmic plots for fluorescence quenching of BP by GA and CAF.

UV–visible absorption spectroscopy is widely used to explore the structural changes of protein and to investigate protein–ligand complex formation [42]. Accordingly, the UV–Vis analysis in this study focused on the 230–360 nm region, which contains the main informative absorption band for evaluating microenvironmental changes in the BP system. As shown in Figure 6A, upon adding PEC to BSA, the absorbance of the aromatic band at 278 nm increased with increasing pectin concentration, while the peak position remained nearly unchanged, indicating that pectin only induces limited perturbation to the microenvironment of BSA aromatic residues; therefore, BP can be regarded as a relatively stable “baseline complex” for subsequent titrations [43]. On this basis, Figure 6B shows that after GA was introduced into the BP complex, the UV spectrum exhibited a noticeable red shift, which is commonly interpreted as evidence that GA forms a ground-state association/complex with BP and alters the local electronic environment of aromatic residues. In contrast, in Figure 6C, the absorbance in the 240–290 nm region increased more dramatically after CAF addition, but the red shift was not obvious; considering that CAF itself has strong UV absorption [44], the pronounced spectral changes in Figure 6C are more likely dominated by intrinsic absorbance additivity and spectral overlap. Overall, GA appears to induce a more pronounced conformational/microenvironmental alteration of BSA within the BP complex.

These results suggest that GA and CAF played different roles in the BP system. GA was more likely to induce a real microenvironmental or conformational adjustment in the protein-containing complex, whereas the UV response after CAF addition was more strongly affected by its own intrinsic absorbance. This difference provides an important clue for understanding why GA may be more favorable for stable incorporation into the nanoparticle matrix, while CAF may be less effective in promoting a more organized colloidal assembly.

#### 3.6.2. Fluorescence Evidence for Distinct Local Interactions of GA and CAF with the BP Complex

Fluorescence spectroscopy is widely used to investigate protein microenvironmental changes and protein–ligand interactions [45]. Both GA and CAF decreased the intrinsic fluorescence intensity of the BP complex in a concentration-dependent manner (Figure 6D,E). Specifically, GA reduced the fluorescence intensity of BP from 8608 to 1750, whereas CAF reduced it further to 1327, indicating that both compounds interact with BP and induce fluorescence quenching. Meanwhile, both GA and CAF quench BP intrinsic fluorescence in a concentration-dependent manner (Figure 6D,E). The Stern–Volmer plots (Figure 6F) are well-described by Equation (11):

(11) $$\frac{F_{0}}{F}=1+K_{SV}\left[Q\right]$$

where *F*_0_ and *F* denote the fluorescence intensity in the absence and presence of quencher, respectively, [*Q*] is the concentration of quencher, and *K_SV_* is the Stern-Volmer quenching constant. According to the results, *K_SV_* = 7.6 × 10^6^ L/mol for GA and 1.11 × 10^7^ L/mol for CAF, indicating a higher apparent quenching efficiency for CAF.

The double-logarithmic analysis (Figure 6G) follows Equation (12):

(12) $$\mathrm{lg}\left(\frac{F_{0}}{F}-1\right)=lgK_{a}+nlg\left[Q\right]$$

where *K_a_* is the binding constant, *n* is the number of binding sites. The values of lg*K_a_* and *n* can be obtained from the intercept and the slope of the double logarithm regression curve. According to the results, the values of *n* were approximate to 1, indicating approximately a single dominant binding site in this concentration range. The corresponding calculated *K_a_* between GA or CAF and BSA were 1.43 × 10^6^ L/mol and 5.59 × 10^7^ L/mol. These results indicate that CAF exhibited a stronger local quenching capability toward the fluorescence-sensitive groups in the BP complex.

However, stronger fluorescence quenching does not necessarily mean that CAF was more effective in stabilizing the BP assembly as a whole. Fluorescence mainly reflects changes in the local environment around fluorescent residues, whereas nanoparticle stabilization depends on whether the added molecule can promote a more cooperative and organized intermolecular network. In this context, the fluorescence results should be interpreted together with the UV data. As discussed above, GA produced a clearer red shift in the BP system, which is more consistent with a distinct ground-state association and a real microenvironmental rearrangement. In contrast, the UV response of CAF was more strongly influenced by its intrinsic absorption overlap. Therefore, although CAF showed a stronger apparent quenching effect, the combined spectroscopic evidence suggests that GA was more effective in inducing a structurally meaningful reorganization of the BP complex.

This distinction is important for understanding the compositional remodeling observed in WTMPs. A molecule that produces strong local quenching is not necessarily the one that best supports stable incorporation into a protein–polysaccharide–polyphenol network. Instead, the results suggest that GA was more likely to act as a cooperative assembly participant in the BP system, whereas CAF may have interacted more locally with fluorescence-sensitive regions without contributing equally to network stabilization. This interpretation is consistent with the higher GA content and lower CAF content observed in WTMPs after optimized isolation, and it provides an important mechanistic link between selective component retention and improved nanoparticle stability.

#### 3.6.3. Circular Dichroism Spectroscopy Analysis

**Figure 7:**
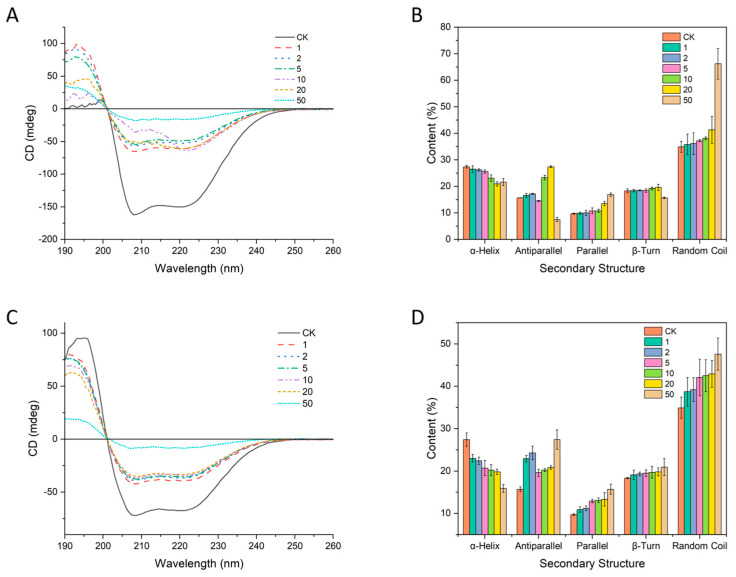
(**A**) Effects of GA on the circular dichroism spectra of the BP complex. (**B**) Effects of GA on the secondary structure contents of BSA in the BP complex. (**C**) Effects of CAF on the circular dichroism spectra of the BP complex. (**D**) Effects of CAF on the secondary structure contents of BSA in the BP complex.

As shown in Figure 7A, the BP complex (CK) displayed the characteristic α-helical double minima near 208 and 222 nm in the far-UV region, indicating that BSA in BP retains a predominantly helix-rich, ordered secondary structure. With increasing GA concentration, the negative ellipticity at 208 and 222 nm progressively decreased and the spectrum became markedly flattened at high levels, which is a typical spectral signature of substantial weakening of helical order and a shift toward more disordered conformations. Consistently, the deconvoluted secondary-structure contents in Figure 7B showed a decrease in α-helix from 27.4% to 20.9%, accompanied by a pronounced increase in random coil from 34.9% to 66.2%, while β-turn changes remain comparatively modest, suggesting that GA primarily drives a helix-to-disorder transition. Such percentage estimates are commonly obtained through CD spectral deconvolution, enabling robust relative comparisons across treatments. In contrast, under CAF treatment (Figure 7C), the helical minima also weaken, yet the overall spectral profile tends to retain more helix-like features across much of the range; correspondingly, Figure 7D indicates that α-helix decreased from 27.4% to 15.9% alongside increases in β-associated components (antiparallel from 15.7% to 27.4% and parallel from 9.7% to 15.7%), whereas the rise in random coil was comparatively less dramatic than for GA from 34.9% to 47.6%.

These differences are important for understanding the distinct roles of GA and CAF in the BP system. Both molecules altered the secondary structure of BSA, but the mode of restructuring was not the same. GA caused a more pronounced loss of helical order and a stronger shift toward disordered conformations, which is consistent with a larger-scale conformational rearrangement of the protein-containing complex. In contrast, CAF also reduced the α-helical content, but its effect was more closely associated with a redistribution toward β-related structures. When considered together with the UV and fluorescence results, the CD data further suggest that GA was more effective in driving a structurally meaningful reorganization of the BP complex. This provides additional support for the idea that GA is more favorable for cooperative assembly within the nanoparticle matrix, whereas CAF is more likely to produce a more local or differently organized interaction pattern.

### 3.7. Molecular Dynamics Simulations

To further explain why GA was enriched whereas CAF decreased in WTMPs after optimized isolation, molecular dynamics simulations were performed on ternary model systems containing BSA, PEC, and either GA or CAF. Although a single model protein cannot fully represent the complexity of native tea proteins, BSA is a well-characterized globular protein with a resolved three-dimensional structure and well-studied polyphenol binding behavior. It has therefore been widely used to model protein–polyphenol and protein–polysaccharide interactions in food systems [12,46]. PEC was selected as the representative polysaccharide because the FTIR results suggested that PEC-related structures were present in both WTCs and WTMPs. GA and CAF were selected because both were identified in the compositional analysis, and their contents changed in opposite directions after optimized isolation. Therefore, the BP, BPG, and BPC systems provided a simplified but useful framework for examining how different tea small molecules may influence ternary assembly stability. However, this model should be interpreted as a simplified mechanistic system for probing relative interaction tendencies, rather than as a direct molecular representation of the native tea-protein network in white tea infusions [47].

#### 3.7.1. Structural Stability and Interfacial Organization of BPG and BPC

**Figure 8:**
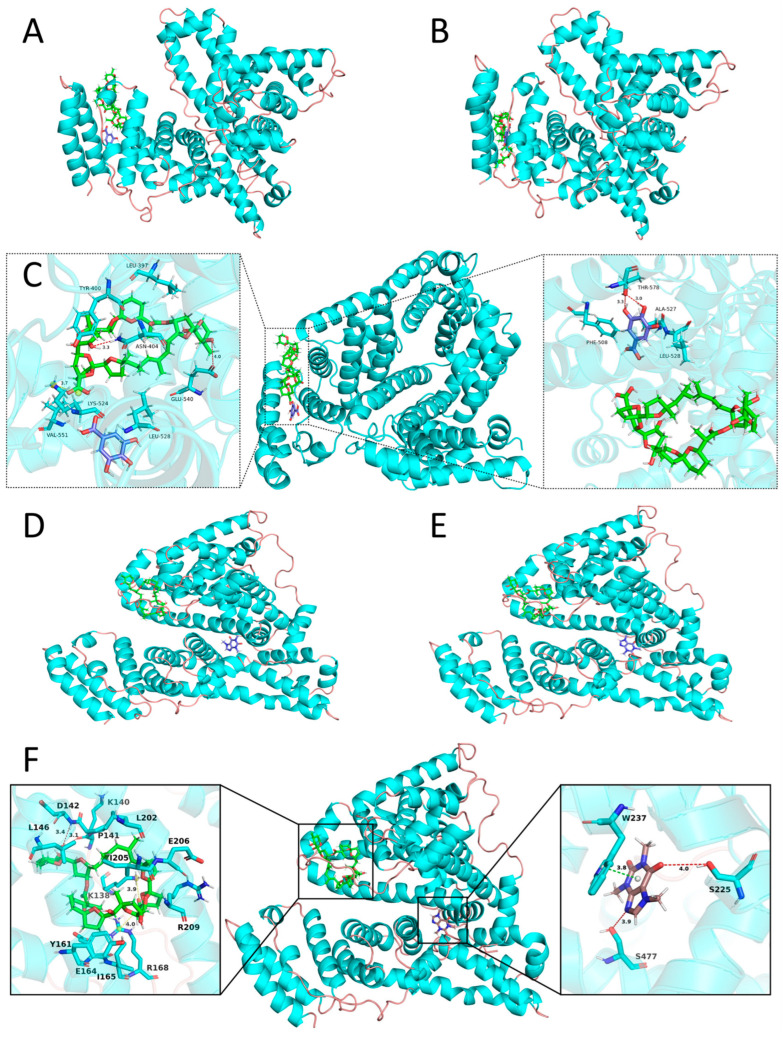
(**A**) Structural rendering of the BPG complex at 0 ns (green sticks: PEC, purple sticks: GA, blue cartoon: protein, light pink regions: protein coils, same below); (**B**) structural rendering of the BPG complex at 100 ns; (**C**) conformation and interactions of the BPG ternary complex at 100 ns; (**D**) structural rendering of the BPC complex at 0 ns (brown sticks: CAF, same below); (**E**) structural rendering of the BPC complex at 100 ns; (**F**) conformation and interactions of the BPC ternary complex at 100 ns.

Figure 8 suggests that GA plays an interfacial “bridging” role that is more stabilizing for the ternary assembly than CAF. In the BPG system, GA (purple) remains co-localized with PEC (green) on the protein surface from 0 ns to 100 ns (Figure 8B), and the 100 ns close-up (Figure 8C) shows multiple short contacts consistent with hydrogen-bonding and tight polar/π interactions between GA and nearby residues while GA stays adjacent to the PEC chain; this geometry is best interpreted as a multidentate interfacial anchor that increases the connectivity of the B–P interface like a “B–G–P” bridge. Such polyphenol-mediated interfacial cross-linking is widely recognized to enhance the integrity and kinetic stability of protein–polysaccharide assemblies by adding extra interaction nodes and reducing interfacial mobility [9]. In contrast, in the BPC system, CAF (brown) appears primarily as a pocket/near-pocket ligand whose local contacts at 100 ns (Figure 8F, like interactions with W237/S225/S477) do not visibly connect the PEC chain to the protein surface; instead, the B–P framework is maintained mainly by direct BSA–PEC contacts (Figure 8F). Given that CAF-BSA binding is commonly described as relatively weak-to-moderate and readily exchangeable compared with multidentate polyphenols, CAF is less likely to serve as a structural node in a ternary scaffold. Therefore, even though CAF can bind albumin, the structural evidence in Figure 8 favors higher ternary stability for BPG than BPC because GA increases the interfacial cross-link density (bridge-like binding), whereas CAF mainly contributes local pocket occupancy without reinforcing the B–P interface. This interpretation is also consistent with the broader literature on phenolic acids forming stabilizing, multi-contact complexes with serum albumins [9].

This interpretation is also consistent with the experimental results presented above. The compositional analysis showed that GA was enriched whereas CAF decreased in WTMPs after optimized isolation. The spectroscopic results further showed that GA induced a more interpretable microenvironmental and conformational rearrangement in the BP system, while CAF showed stronger local quenching but a less clearly stabilizing structural effect. The MD results now provide a direct structural explanation for these observations. They suggest that GA is more favorable for stable incorporation into the protein–polysaccharide–polyphenol framework of WTMPs, whereas CAF is less likely to serve as a stabilizing structural node in the nanoparticle matrix.

#### 3.7.2. RMSD Analysis

**Figure 9:**
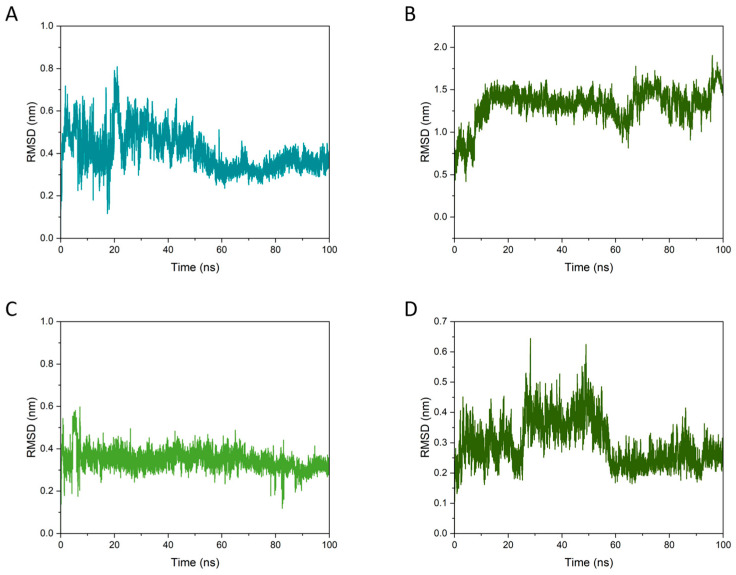
(**A**) RMSD curve of GA relative to BSA in BPG complex; (**B**) RMSD curve of PEC relative to BSA in BPG complex; (**C**) RMSD curve of CAF relative to BSA in BPC complex; (**D**) RMSD curve of PEC relative to BSA in BPC complex.

The RMSD curves reflected the root mean square deviation of the two molecules relative to the protein during the simulation and were used in this study to evaluate the stability of their binding to BSA [48]. Figure 9 provides dynamic evidence that GA more effectively cooperates with PEC to stabilize the ternary BPG assembly, whereas CAF behaves primarily as a locally bound ligand with limited interfacial reinforcement. In the BPG system, the ligand RMSD of GA relative to BSA (Figure 9A) fluctuates during the early stage but converges to a lower, sustained plateau after 50–60 ns, indicating that GA relaxes into a more persistent bound pose (RMSD plateaus are commonly used as a practical indicator of equilibration/stationary behavior in MD trajectories). In parallel, the pectin RMSD relative to BSA in BPG (Figure 9B) rapidly increases within the first 10–20 ns and then remains largely stationary around a broad plateau about 1.5 nm. For a highly flexible polysaccharide chain, such patterns are most consistent with early reorientation/repositioning on the protein surface followed by stable residence in a new interfacial configuration, rather than progressive dissociation [49].

In contrast, in the BPC system, the RMSD of CAF relative to BSA (Figure 9C) stayed at a relatively low and narrow range, consistent with a stable pocket-proximal binding pose; ligand RMSD in this range is frequently used as a qualitative sign of pose retention in protein–ligand MD analyses. However, the pectin RMSD relative to BSA in BPC (Figure 9D) showed a more pronounced two-regime behavior, with an evident elevation and larger spikes around 30–55 ns followed by a drop to a lower plateau. This delayed transition suggests less synchronized interfacial stabilization, which means that PEC requires a later rearrangement to reach a stable configuration, consistent with the structural snapshots (Figure 8) where GA adopts an interfacial “bridge-like” geometry that can increase the connectivity of the B–P interface, while CAF mainly contributes local interactions without directly cross-linking the BSA–PEC interface. Such polyphenol-mediated multidentate interactions are widely discussed as a stabilizing mechanism in protein–polysaccharide–polyphenol ternary assemblies [50].

Overall, the RMSD results provide dynamic evidence that BPG reached a more coordinated and persistent interfacial state than BPC over the 100 ns timescale. This finding further supports the conclusion that GA was more effective than CAF in promoting cooperative stabilization of the protein–polysaccharide–polyphenol assembly. More importantly, it helps explain why GA was more readily retained, whereas CAF was reduced, in the optimized WTMP fraction. Together with the compositional, spectroscopic, and morphological results above, the MD analysis supports a consistent mechanism in which GA acts as a stabilizing interfacial participant, while CAF behaves mainly as a locally bound but less structure reinforcing molecule.

#### 3.7.3. Number of Hydrogen Bonds Formed

**Figure 10:**
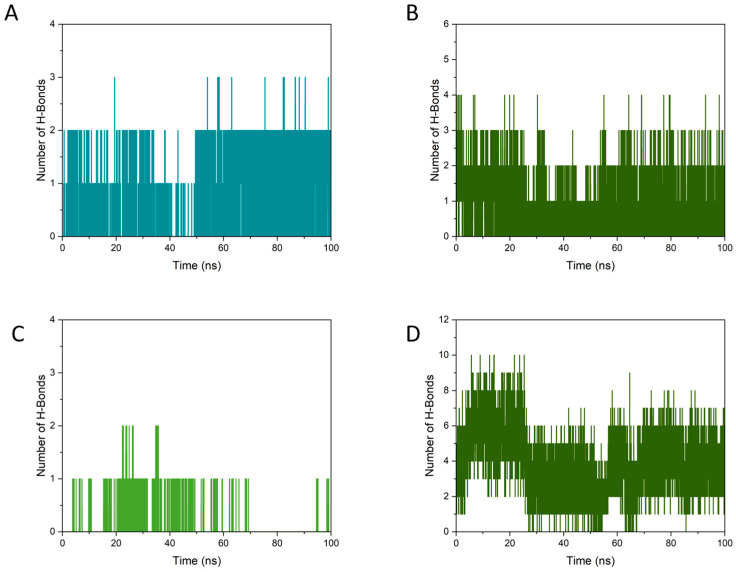
(**A**) Number of hydrogen bonds formed between BSA and GA in BPG. (**B**) Number of hydrogen bonds formed between BSA and PEC in BPG. (**C**) Number of hydrogen bonds formed between BSA and CAF in BPC. (**D**) Number of hydrogen bonds formed between BSA and PEC in BPC.

In MD simulations, hydrogen bonds are generally considered far more significant than other types of interactions, particularly since hydrogen bond interactions are dominant contributors to electrostatic interactions. Thus, they are regarded as one of the most important driving forces in ligand binding [51]. Figure 10 quantifies the hydrogen-bond (H-bond) network underpinning the BSA–polysaccharide–small-molecule assemblies and highlights a key mechanistic difference between GA and CAF. In the BPG system, GA forms persistent but moderate H-bonding with BSA (Figure 10A), remaining predominantly in the 1–2 H-bond range with occasional excursions to higher counts, and showing an apparent increase in sustained contacts after the mid-trajectory region behavior consistent with a ligand that relaxes into a stable interfacial pose while retaining continuous polar anchoring. This pattern is aligned with prior modeling and spectroscopy studies reporting that GA establishes specific H-bonds with albumin binding pockets and maintains stable association during MD [52]. Meanwhile, the BSA–PEC H-bonds in BPG (Figure 10B) fluctuate around a moderate plateau (commonly 2–3 with transient decreases), which is expected for a flexible polysaccharide adapting on a protein surface; importantly, the presence of GA adds an additional, continuously present H-bond contribution that effectively increases the interfacial connectivity of the ternary complex, a mechanism widely discussed for polyphenol-mediated stabilization of protein–polysaccharide assemblies [53].

In the BPC system, CAF exhibited scarce H-bonding to BSA (Figure 10C), with the trajectory dominated by 0–1 H-bond events and only sporadic higher counts, indicating that CAF contributes little to the polar cross-linking of the ternary scaffold and is more consistent with an exchangeable pocket/near-pocket ligand whose binding is often dominated by van der Waals interactions with limited H-bond support. In contrast, BSA–PEC H-bonding in BPC (Figure 10D) was substantially higher at frequently 3–8, yet it also displayed a pronounced mid-trajectory regime shift, implying that interfacial stabilization relies primarily on BSA–PEC rearrangement rather than cooperative reinforcement by the small molecule. Taken together, Figure 10 supports the interpretation that GA acts as a cooperative “interfacial stabilizer”, providing persistent BSA–GA H-bonds in addition to the BSA–PEC network, whereas CAF contributes minimally to hydrogen-bond cross-linking, making the BPC ternary architecture more dependent on the prBSA–PEC interface alone. This is consistent with the broader literature emphasizing that polyphenols can increase ternary-complex robustness by adding multidentate interaction nodes and strengthening interfacial networks.

These hydrogen-bond results further clarify the different roles of GA and CAF in the ternary systems. In BPG, GA does not simply bind to BSA. Instead, it provides an additional and persistent polar interaction layer on top of the BSA–PEC network, which helps maintain interfacial connectivity throughout the trajectory. In BPC, CAF contributes far less to hydrogen-bond cross-linking, so the stability of the assembly depends more strongly on the BSA–PEC interface alone. This difference is fully consistent with the structural and RMSD results above. More importantly, it helps explain the compositional remodeling observed in WTMPs after optimized isolation. GA was more likely to be retained because it could participate in cooperative interfacial stabilization, whereas CAF was less able to function as a stabilizing structural node. Therefore, the hydrogen-bond analysis provides another layer of evidence that the improved stability of WTMPs was associated not only with protein–polysaccharide contacts, but also with the selective incorporation of small molecules, especially GA, which reinforced the ternary interaction network.

#### 3.7.4. SASA and Secondary Structure Evolution

This study further analyzed the variation in the solvent accessible surface area (SASA) of the protein during the simulation. SASA reflects, to some extent, the folding tendency of the protein and mainly indicates the degree of molecular burial. A larger SASA value corresponds to greater surface exposure and a larger contact area with the solvent, whereas a smaller SASA value indicates stronger burial and reduced solvent accessibility [54].

**Figure 11:**
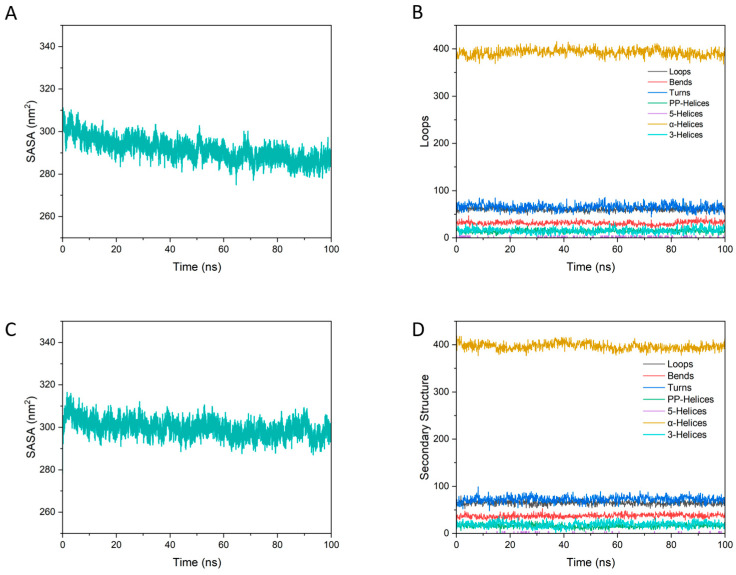
(**A**) SASA variation trend of BPG. (**B**) Variations in secondary structure content of BPG. (**C**) SASA variation trend of BPC. (**D**) Variations in secondary structure content of BPC.

Overall, Figure 11 indicates that both ternary complexes remained conformationally stable over 100 ns, while the BPG system showed a stronger tendency toward surface compaction without secondary-structure disruption, consistent with GA acting as a cooperative stabilizer of the BSA–PEC scaffold. Specifically, the SASA trace of BPG (Figure 11A) exhibited a gradual decrease from an initially higher value to a lower plateau at about 250 nm^2^ toward the end of the trajectory, suggesting reduced solvent exposure and progressive compaction of the assembled structure. In contrast, the SASA trace of BPC (Figure 11C) stabilized earlier and fluctuated around a comparatively higher level, implying a less pronounced net compaction on the same timescale [48]. Consistently, the secondary-structure profiles for BPG (Figure 11B) and BPC (Figure 11D) showed only small-amplitude fluctuations across structural classes with no persistent drift indicative of large-scale unfolding; this supports that the protein scaffold remains structurally intact, which is important because serum albumin is known to possess a robust, helix-rich fold whose global secondary structure is typically resilient unless strongly perturbed.

These results provide another layer of support for the different roles of GA and CAF in the ternary assemblies. In BPG, GA promoted a more compact interfacial architecture while preserving the overall fold of the protein framework. In BPC, the system also remained stable, but the degree of compaction was weaker, which is more consistent with CAF acting as a locally associated ligand than as an effective interfacial stabilizer. This distinction agrees well with the compositional, spectroscopic, and other MD results presented above. More importantly, it helps explain why GA was more favorable for retention in WTMPs and why its enrichment was associated with improved nanoparticle stability after optimized isolation.

#### 3.7.5. MM/GBSA Energy Decomposition and Interaction Mechanism

In this study, the binding free energies of the complexes were evaluated using the MM/GBSA method based on the MD trajectories. MM/GBSA is widely used to estimate binding free energy and to decompose it into van der Waals, electrostatic, and solvation terms, thereby helping to interpret the driving forces of molecular interactions [55].

**Figure 12:**
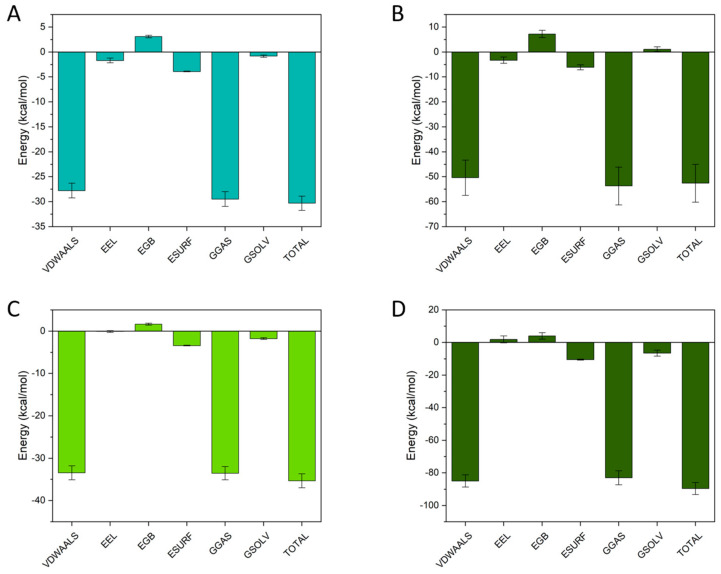
(**A**) MM/GBSA energy decomposition of GA in BPG; (**B**) MM/GBSA energy decomposition of PEC in BPG; (**C**) MM/GBSA energy decomposition of CAF in BPC; (**D**) MM/GBSA energy decomposition of PEC in BPC.

Combining Figure 12 with Table 3 shows that binding in both ternary systems is dominated by nonpolar/dispersion-driven interactions, while the role of the small molecule differs markedly in whether it strengthens the BSA–PEC interface. For GA in BPG (Figure 12A; Table 3), the total binding free energy was Δ*E*_Bind_ = −30.31 kcal/mol, largely arising from van der Waals interactions (Δ*E*_vdw_ = −27.76 kcal/mol), with only a minor electrostatic contribution; solvation was nearly neutral but slightly favorable because the unfavorable polar term (Δ*E*_GB_ = 3.07 kcal/mol) is partially offset by a favorable nonpolar surface term (Δ*E*_surf_ = −3.91 kcal/mol). In contrast, CAF in BPC binds somewhat more strongly to BSA (Figure 12C), again driven primarily by van der Waals (Δ*E*_vdw_ = −33.45 kcal/mol) with negligible electrostatics and slightly more favorable solvation.

Critically, the scaffold interaction BSA–PEC was far stronger in the CAF system than in the GA system: PEC binding to BSA was −89.6 kcal/mol in BPC (Figure 12D; Table 3) versus −52.6 kcal/mol in BPG (Figure 12B; Table 3), and the difference was overwhelmingly explained by the much more favorable van der Waals term for pectin in BPC (−84.95 vs. −50.37 kcal/mol), together with a more favorable nonpolar solvation term, despite an unfavorable electrostatic term for pectin in BPC. This pattern—strongly negative van der Waals plus nonpolar solvation dominating bind energy—is typical for end-point MM/GBSA decompositions and supports interpretation in terms of hydrophobic/dispersion-driven interfacial packing [24].

However, these energy values should not be interpreted in isolation. A stronger local binding energy does not necessarily mean a higher level of cooperative stabilization of the ternary assembly. When the MM/GBSA results are considered together with the RMSD, hydrogen-bond, and SASA analyses, a clearer picture emerges. CAF could interact strongly with BSA, but this interaction was dominated mainly by local van der Waals contacts and contributed less to persistent interfacial reinforcement. In contrast, GA showed slightly weaker direct binding to BSA, yet it was more effective in providing polar anchoring and in participating in the interfacial organization of the BSA–PEC assembly. In this sense, GA functioned less as a simple ligand and more as a cooperative structural participant.

This distinction is central to understanding the stability of WTMPs. The results suggest that stable ternary assembly depends not only on how strongly a small molecule binds to the protein, but also on whether it can reinforce the interaction network at the protein–polysaccharide interface. Therefore, even though CAF–BSA binding appeared somewhat stronger in MM/GBSA, the combined structural, dynamic, and energetic evidence still supports BPG as the more cooperatively stabilized ternary system. This interpretation is fully consistent with the experimental findings above, especially the enrichment of GA, the reduction in CAF, and the improved environmental stability of WTMPs after optimized isolation.

#### 3.7.6. Mechanistic Summary of Molecular Dynamics Results

The molecular dynamics results consistently suggested that GA and CAF played different roles in the ternary assemblies. In the BPG system, GA behaved more like an interfacial participant than a simple bound ligand. It contributed to persistent interfacial contacts, more coordinated dynamic stabilization, and a more compact assembly without disrupting the overall protein fold. In contrast, CAF in the BPC system was more consistent with a pocket or near-pocket ligand. Although it could remain locally associated with BSA and even showed relatively strong local binding, its contribution to cooperative stabilization of the BSA–PEC interface was weaker.

This distinction helps explain the compositional remodeling and stability enhancement observed in WTMPs after optimized isolation. Stable nanoparticle formation depends not only on whether a small molecule can bind to the protein, but also on whether it can reinforce the interaction network of the ternary assembly. In this respect, GA was more favorable than CAF. Therefore, the MD results provide molecular-level support for the interpretation that GA enrichment was associated with, and may contribute to, the improved stability of WTMPs.

Taken together, the present results support a composition–structure–stability relationship for WTMPs. The optimized ultrafiltration–centrifugation process did not merely concentrate the parent tea colloids but selectively enriched a particle fraction with remodeled composition, particularly higher GA retention and lower CAF content. This compositional shift was associated with more regular morphology, more ordered intermolecular organization, and improved resistance to environmental perturbations. The spectroscopic and MD results further suggest that GA was more favorable for cooperative interfacial stabilization within the protein–polysaccharide network, whereas CAF behaved more like a locally associated ligand with weaker contribution to network reinforcement. Accordingly, the enhanced stability of WTMPs is more reasonably interpreted as being associated with selective compositional remodeling and strengthened interfacial organization, rather than with a simple concentration effect alone.

## 4. Conclusions

This study established a stability-oriented strategy for isolating white tea micro-nanoparticles and clarified their stabilization mechanism from a composition–structure–stability perspective. The optimal condition was identified as a 100 kDa membrane, 3000× *g*, and 20 min, showing that effective WTMP isolation depends on balancing particle retention, purification, and colloidal stability rather than optimizing a single parameter.

Under the optimized condition, the isolated WTMPs were not a simple concentrate of the original tea colloids. Instead, they represented a selectively enriched nanoparticle fraction with remodeled composition, more regular morphology, more ordered intermolecular organization, and stronger environmental stability. In particular, GA was enriched whereas CAF decreased, suggesting that small-molecule retention in WTMPs was selective rather than random.

The mechanistic analyses further supported the interpretation that GA and CAF may play different roles in the protein–polysaccharide–polyphenol network. Spectroscopy and molecular dynamics simulations consistently indicated that GA was more favorable for cooperative interfacial stabilization, whereas CAF behaved more like a locally associated ligand with weaker contribution to network reinforcement. This difference provides a molecular explanation for the selective enrichment of GA and the enhanced stability of WTMPs. Although the present results support a mechanistic association between GA enrichment and improved stability, direct causal validation will require additional targeted experiments in future work.

Overall, this study integrates optimized isolation, structural characterization, and mechanistic analysis into a coherent framework for understanding WTMP stability. It shows that the improved stability of WTMPs arises not only from purification, but also from selective compositional remodeling and more effective interfacial organization. These findings provide a theoretical basis for improving white tea colloidal quality and developing stable white tea-based functional products.

## Figures and Tables

**Table 1:** Final ranking derived by the TOPSIS method across MWCO, centrifugation times, and centrifugal forces.

MWCO (kDa)	Time (min)	Force (g)	d^+^	d^−^	C	Ranking
100	20	3000	0.0262	0.0746	0.7404	1
100	10	5000	0.0271	0.0700	0.7211	2
100	10	4000	0.0269	0.0684	0.7176	3
100	20	4000	0.0295	0.0739	0.7148	4
100	20	5000	0.0315	0.0772	0.7101	5
100	30	4000	0.0373	0.0793	0.6804	6
100	10	6000	0.0341	0.0694	0.6702	7
100	10	3000	0.0316	0.0629	0.6654	8
100	20	6000	0.0401	0.0777	0.6597	9
100	30	5000	0.0419	0.0766	0.6462	10
100	30	6000	0.0412	0.0733	0.6403	11
100	30	3000	0.0451	0.0735	0.6200	12
100	30	2000	0.0452	0.0712	0.6117	13
100	20	2000	0.0440	0.0679	0.6065	14
30	10	6000	0.0412	0.0611	0.5969	15
30	20	6000	0.0404	0.0571	0.5856	16
30	20	5000	0.0422	0.0527	0.5551	17
100	10	2000	0.0440	0.0542	0.5522	18
30	30	5000	0.0484	0.0541	0.5277	19
30	30	6000	0.0529	0.0590	0.5270	20
30	10	5000	0.0509	0.0550	0.5191	21
30	20	4000	0.0536	0.0397	0.4254	22
30	10	4000	0.0627	0.0425	0.4039	23
30	30	4000	0.0591	0.0354	0.3750	24
30	30	3000	0.0627	0.0326	0.3423	25
30	20	3000	0.0663	0.0296	0.3090	26
30	30	2000	0.0689	0.0301	0.3043	27
30	10	3000	0.0688	0.0295	0.3000	28
30	20	2000	0.0764	0.0239	0.2380	29
30	10	2000	0.0840	0.0255	0.2326	30

**Table 2:** Concentrations of major components in WTCs and WTMPs (μg/mg). Different lowercase letters represent a significant difference at *p* < 0.05.

Component	WTCs	WTMPs	*p* Value
Protein	379 ± 14 ^b^	412 ± 12 ^a^	0.037
Polysaccharide	114 ± 5 ^a^	109 ± 2 ^a^	0.219
Tea polyphenol	427 ± 2 ^a^	386 ± 3 ^b^	1.1 × 10^−4^
Caffeine	91 ± 0.1 ^a^	62 ± 0.1 ^b^	3.77 × 10^−10^
GA	10 ± 0.2 ^b^	39 ± 0.1 ^a^	2.54 × 10^−7^

**Table 3:** Decomposition of binding energy components of PEC and GA with BSA (kcal/mol).

Energy	GA in BPG	PEC in BPG	CAF in BPC	PEC in BPC
Δ*E*_vdw_	−27.76	−50.37	−33.45	−84.95
Δ*E*_ele_	−1.71	−3.31	−0.1	1.89
Δ*E*_GB_	3.07	7.21	1.64	4.01
Δ*E*_surf_	−3.91	−6.12	−3.41	−10.55
Δ*E*_Gas_	−29.47	−53.68	−33.55	−83.06
Δ*E*_solv_	−0.84	1.08	−1.77	−6.55
Δ*E*_Bind_	−30.31	−52.6	−35.32	−89.6

*Note:* Δ*E*_vdw_ represents van der Waals energy; Δ*E*_ele_ represents electrostatic energy; Δ*E*_Gas_ represents gas-phase free energy, where Δ*E*_Gas_ = Δ*E*_vdw_ + Δ*E*_ele_; Δ*E*_surf_ represents nonpolar solvation energy; Δ*E*_GB_ represents polar solvation energy; Δ*E*_solv_ represents solvation free energy, where Δ*E*_solv_ = Δ*E*_GB_ + Δ*E*_surf_; Δ*E*_Bind_ denotes the total binding free energy, calculated as Δ*E*_Bind_ = Δ*E*_Gas_ + ΔE_solv_.

## Data Availability

The original contributions presented in the study are included in the article. Further inquiries can be directed to the corresponding author.

## Abbreviations

The following abbreviations are used in this manuscript:

WTC	White tea colloid
WTMP	White tea infusion micro-nanoparticles
RMSD	Root mean square deviation
SASA	Solvent-accessible surface area
MM/GBSA	Molecular mechanics/generalized Born surface area
FTIR	Fourier transform infrared
BSA	Bovine serum albumin
PEC	Pectin
GA	Gallic acid
CAF	Caffeine
PDI	Polydispersity index
SEM	Scanning electron microscope
TEM	Transmission electron microscope
TOPSIS	Technique for Order Preference by Similarity to Ideal Solution
CD	Circular dichroism
MD	Molecular Dynamics
